# Unexpectedly high genetic diversity in a rare and endangered seabird in the Hawaiian Archipelago

**DOI:** 10.7717/peerj.8463

**Published:** 2020-02-06

**Authors:** Carmen C. Antaky, Emily E. Conklin, Robert J. Toonen, Ingrid S.S. Knapp, Melissa R. Price

**Affiliations:** 1 Department of Natural Resources and Environmental Management, University of Hawai‘i at Mānoa, Honolulu, HI, USA; 2 Hawai‘i Institute of Marine Biology, University of Hawai‘i at Mānoa, Kāne‘ohe, HI, USA

**Keywords:** Band-rumped Storm Petrel, Endangered species, Inbreeding, Population genetics, Procellariiformes, *Hydrobates castro*, Oceanodroma, Hawai‘i

## Abstract

Seabirds in the order of Procellariiformes have one of the highest proportions of threatened species of any avian order. Species undergoing recovery may be predicted to have a genetic signature of a bottleneck, low genetic diversity, or higher rates of inbreeding. The Hawaiian Band-rumped Storm Petrel (‘Akē‘akē; *Hydrobates castro*), a long-lived philopatric seabird, suffered massive population declines resulting in its listing under the Endangered Species Act in 2016 as federally Endangered. We used high-throughput sequencing to assess patterns of genetic diversity and potential for inbreeding in remaining populations in the Hawaiian Islands. We compared a total of 24 individuals, including both historical and modern samples, collected from breeding colonies or downed individuals found on the islands of Kaua‘i, O‘ahu, Maui, and the Big Island of Hawai‘i. Genetic analyses revealed little differentiation between breeding colonies on Kaua‘i and the Big Island colonies. Although small sample sizes limit inferences regarding other island colonies, downed individuals from O‘ahu and Maui did not assign to known breeding colonies, suggesting the existence of an additional distinct breeding population. The maintenance of genetic diversity in future generations is an important consideration for conservation management. This study provides a baseline of population structure for the remaining nesting colonies that could inform potential translocations of the Endangered *H. castro*.

## Introduction

Many seabird species in the order Procellariiformes have experienced historical bottlenecks due to vulnerability to anthropogenic disturbances (Weimerskirch, Brothers & Jouventin, 1997; Milot et al., 2007; Paleczny et al., 2015). Current management, including predator control, laws protecting seabirds, and habitat restoration are helping to stabilize and restore some of these seabird populations (Young et al., 2009; Jones & Kress, 2012; Young et al., 2013). However, despite this active management, many recovering populations still have relatively low genetic variation (Barrowclough, Corbin & Zink, 1981; Sibley & Ahlquist, 1990; Milot et al., 2007). A severe or sufficiently prolonged population reduction, or bottleneck, is expected to result in a corresponding decline in genetic diversity and individual heterozygosity (Wright, 1938; Lande, 1988). Supporting this theoretical prediction, using a meta-analysis of 170 globally threatened and endangered species, Spielman, Brook & Frankham (2004) showed 131 of species examined (~77%) had significantly lower heterozygosity than their closest non-threatened relative. Thus, recovering populations of species in the order Procellariiformes are likely to have a genetic signature of an historical bottleneck, low genetic diversity, and possible inbreeding, all of which can impact individual fitness and extinction risk (Purvis et al., 2000; Frankham, 2010). Nevertheless, not all threatened or endangered species show reduced genetic diversity (Spielman, Brook & Frankham, 2004), and some other animal species are capable of population growth despite dramatically reduced heterozygosity (Schultz et al., 2008). Further, as highly mobile species, Procellariiformes may have mechanisms for maintaining genetic diversity and avoiding inbreeding, such as promiscuous mating behavior and sex-biased dispersal (Milot et al., 2007; Lawrence et al., 2008; Kuro-o et al., 2010).

The Band-rumped Storm Petrel (*Hydrobates castro* or formerly *Oceanodroma castro—*see Wallace et al., 2017 for phylogeny), in the order Procellariiformes, was listed under the Endangered Species Act in 2016, but little is known about its genetic diversity or population connectivity in the Hawaiian Islands (United States Fish and Wildlife Service, 2016). Storm-petrels are relatively long-lived considering their small body size, with most living to 15–20 years. Although unknown for the Hawai‘i populations, age of first breeding in *H. castro* occurs at 5 years in Galápagos and 7 years in Salvages (Harrison, 1990). Once widespread along the Hawaiian Island chain, as evidenced by midden sites on the Main Hawaiian Islands (Harrison, 1990), this species’ breeding range is now limited (Olson & James, 1982; Raine et al., 2017). Due to low population numbers and remote nesting locations, only a few active nests at a single breeding location on the Big Island of Hawai‘i have been confirmed (Galase, 2019; Antaky, Galase & Price, 2019), although they are believed to likely be nesting in small numbers on other Hawaiian islands (Kaua‘i, Maui Nui, and Lehua Islet) based on observational, bycatch, and acoustic data (Pyle & Pyle, 2017). *H. castro* is commonly found in the fossil record across nearly all Southeastern Hawaiian Islands (Harrison, 1990), indicating a larger historic population size before the introduction of mammalian predators (Pyle & Pyle, 2017). As recent evidence of predation by cats and rats was found on the Big Island colony (Galase, 2019), mammalian predators continue to be a large potential threat to existing populations.

Recent studies have produced global phylogenies of *H. castro* in the Atlantic and Pacific Oceans (Smith et al., 2007; Deane, 2013; Taylor et al., 2019), but these large-scale surveys only included samples from Kaua‘i island within the Hawaiian Archipelago. These studies found that the Kaua‘i population is most closely related to the Japan *H. castro* population, but is genetically distinct (Smith et al., 2007; Deane, 2013; Taylor et al., 2019). Therefore, gene flow is likely very low between Hawaiian colonies and populations outside of Hawai‘i (Smith et al., 2007; Taylor et al., 2019). Allochronic populations of *H. castro* have been found elsewhere (Monteiro & Furness, 1998; Deane, 2013), but they do not appear to be present in Hawai‘i (Raine et al., 2017). With only a few hundred breeding pairs suspected to remain (Pyle & Pyle, 2017), there is concern that the Hawaiian populations may have problems normally associated with small population size, such as demographic stochasticity and inbreeding (Brook et al., 2002; Frankham, 2005; Kennedy, 2009). Furthermore, as a member of the Procellariiformes order they are likely highly philopatric (Milot et al., 2007; Jones & Kress, 2012; Antaky et al., in press), with a high rate of return to the natal colony for breeding, potentially contributing to limited gene flow.

Given these pressing management concerns, as a first step in assessing the vulnerability of the remaining populations to a changing environment, in this study we evaluated patterns of genetic variation in Band-rumped Storm Petrels nesting in the Hawaiian Islands. High-throughput sequencing delivers high yields of genetic data across the genome and allows for accurate estimates of genetic relatedness, which is particularly useful for small, potentially inbred populations of non-model organisms. Using high-throughput sequencing, we assessed inbreeding, genetic diversity, genetic structure, and demographic history.

## Materials and Methods

### Sample collection

**Figure 1:**
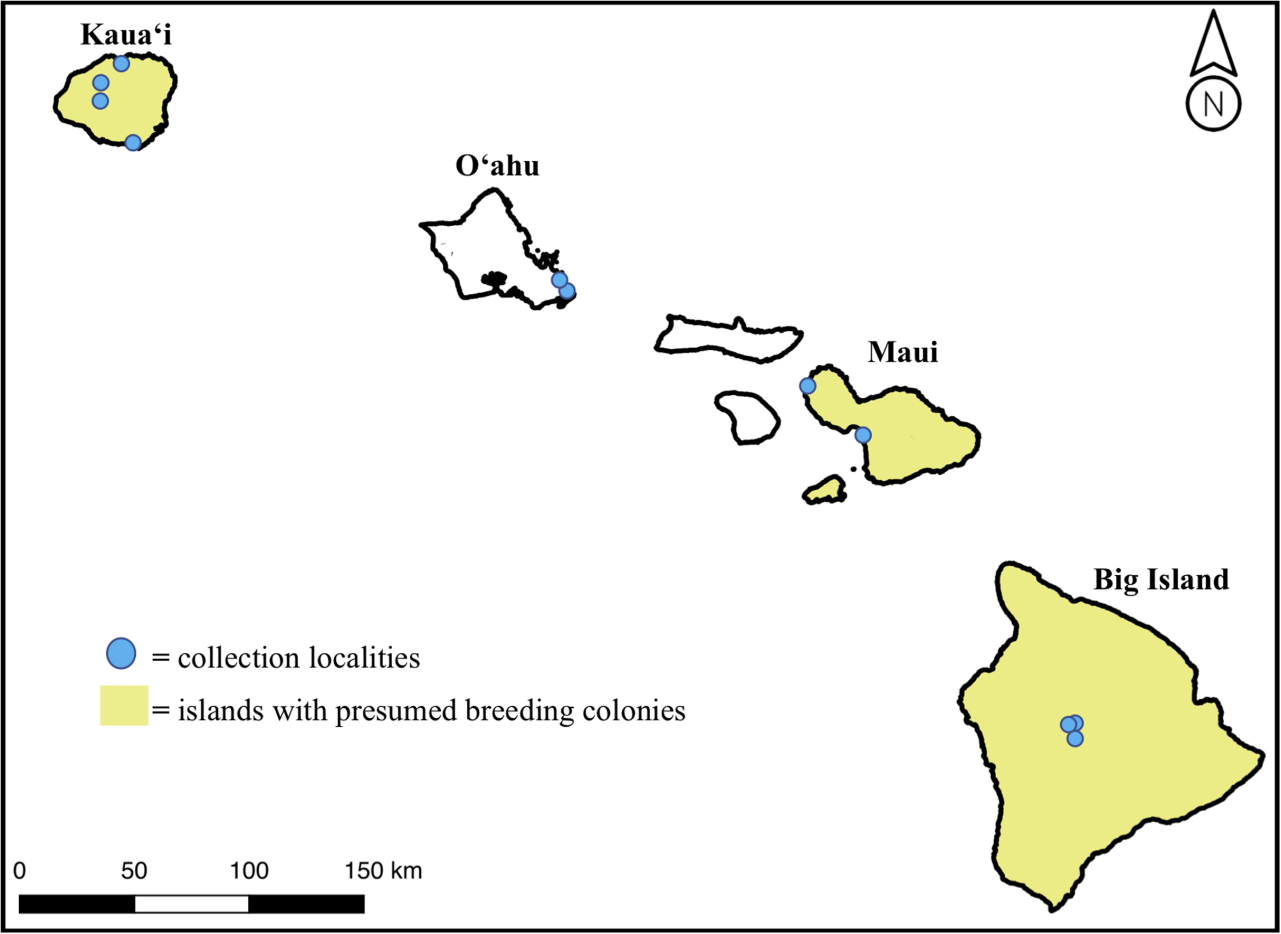
Map of the Main Hawaiian Islands with yellow shading on islands with known or suspected breeding colonies of *Hydrobates castro* (Big Island, Maui Nui, and Kaua‘i). Approximate locations of sampled individuals are marked with a blue circle. Note that the breeding range on each island is not island-wide but represents a lack of knowledge at an island level.

We obtained 18 *H. castro* samples from across the Hawaiian Islands from 4 years of field effort to add to six existing museum specimens, for a total sample size of 24 individuals. Colony-sourced samples came from birds found near suspected or known breeding areas on Kaua‘i, Maui, and the Big Island, while non-colony sourced samples came from the island of O‘ahu (Fig. 1). Samples from O‘ahu were bycatch individuals found on the shoreline on the east side of the island, where there is no known colony. We collected samples under US Fish & Wildlife Permit Number TE25955C-0 and State of Hawaii Department of Land & Natural Resources Protected Wildlife Permit Number WL19-01. Our study was found exempt from Institutional Animal Care and Use Committee (IACUC) protocol review (TEX 16-012).

The Kaua‘i Endangered Seabird Recovery Project (KESRP), a Hawai‘i Department of Land and Natural Resources Division of Forestry and Wildlife project, collected blood samples from the metatarsal vein from individuals on Kaua‘i island between 2014 and 2017 from birds captured using conspecific playback and mist-netting techniques. KESRP stored blood samples on filter paper from one individual in Waimea Canyon, Kaua‘i, five individuals on Honopu Ridge, Kaua‘i, and one from a downed fledgling found in Poipu, Kaua‘i. The Pōhakuloa Training Area (PTA) Natural Resources Office collected samples from individuals at the newly discovered breeding colony on the Big Island between 2015 and 2017, using dog and personnel searches (Galase, 2019). PTA collected flight feathers from nine individual carcasses found near nest sites. Sea Life Park collected two bycatch individuals on O‘ahu island between 2016 and 2017 which were subsequently stored by Dr. David Hyrenbach of Hawai‘i Pacific University. Toe pad and preserved tissue samples collected from specimens at the Bernice Pauahi Bishop Museum included two individuals from the Big Island, two individuals from Maui, and two individuals from Kaua‘i, collected between 1893 and 2008 (Table 1).

**Table 1:** Location information and year of collected tissue and blood samples of *Hydrobates castro* across the Main Hawaiian Islands (*N* = 24).

Island	Collector	Permit #	Year	No. of samples
Kaua‘i	Bernice Pauahi Bishop Museum (CAT#: 156975)	MB675506-0, WL19-21	1893	1
	Bernice Pauahi Bishop Museum (CAT#: 185162)	MB675506-0, WL19-21	2006	1
	Kaua‘i Endangered Seabird Recovery Project	MB673451-0, BBL08487	2014	1
	Kaua‘i Endangered Seabird Recovery Project	MB673451-0, BBL08487	2016	4
	Kaua‘i Endangered Seabird Recovery Project	MB673451-0, BBL08487	2017	2
Kaua‘i subtotal				9
O‘ahu	Dr. David Hyrenbach, Hawai‘i Pacific University	MB180283-0, WL19-01	2016	1
	Dr. David Hyrenbach, Hawai‘i Pacific University	MB180283-0, WL19-01	2017	1
O‘ahu subtotal				2
Maui	Bernice Pauahi Bishop Museum (CAT#: 185001)	MB675506-0, WL19-21	2005	1
	Bernice Pauahi Bishop Museum (CAT#: 185313)	MB675506-0, WL19-21	2008	1
Maui subtotal				2
Big Island	Bernice Pauahi Bishop Museum (CAT#: 183608)	MB675506-0, WL19-21	1994	1
	Bernice Pauahi Bishop Museum (CAT#: 184416)	MB675506-0, WL19-21	2001	1
	Pōhakuloa Training Area Natural Resources Office	MB95880B-0, WL17-10	2015	3
	Pōhakuloa Training Area Natural Resources Office	MB95880B-0, WL17-10	2016	1
	Pōhakuloa Training Area Natural Resources Office	MB95880B-0, WL17-10	2017	5
Big Island subtotal				11
Total				24

### Laboratory analyses

We individually extracted DNA from the blood and feather samples using the DNeasy Blood and Tissue Kit (Qiagen, Valencia, CA, USA) according to the manufacturer’s protocol. We quantified the extracted DNA with the AccuClear™ Ultra High Sensitivity dsDNA Quantitation Kit (Biotium, Hayward, CA, USA). Due to low DNA yield, we performed whole genome amplification on individual samples with the REPLI-g UltraFast Mini-kit (Qiagen, Valencia, CA, USA) which effectively and accurately increases yields of high-fidelity DNA (Ahsanuddin et al., 2017). We prepared the replicated whole genomic DNA from all 24 individuals for reduced representation genomic sequencing using the ezRAD protocol version 3.2 (Toonen et al., 2013; Knapp et al., 2016). In brief, we digested the 24 samples with the frequent cutter restriction enzyme DpnII from New England Biolabs^®^ (Ipswich, MA, USA) and we prepared fragments between 150 and 350 bp in length for sequencing on the Illumina^®^HiSeq using the Kapa Biosystems (Wilmington, MA, USA) Hyper Prep kit with Illumina TruSeq index adapters. We conducted laboratory work at the Hawai‘i Institute of Marine Biology (HIMB) in Kāne‘ohe Bay, O‘ahu, Hawai‘i. We sent libraries to Vincent J. Coates Genomics Sequencing Laboratory at the University of California, Berkeley where they sequenced them on the Ilumina^®^HiSeq 4000 platform with paired-end 2 × 150 bp read length. The raw DNA sequences for each individual were deposited in NCBI Sequence Read Archive (BioProject accession number PRJNA559669).

### Genetic data analyses

We used the dDocent pipeline (Puritz, Hollenbeck & Gold, 2014) to assemble loci and call single nucleotide polymorphisms (SNPs) within the aligned sequences. In dDocent the following settings were used: 90% similarity to cluster reads, match score of one, mismatch score of four, gap penalty of six, minimum coverage of four within individuals, and minimum coverage of three between individuals. We filtered the resulting Variant Call Format (VCF) file using vcftools (Danecek et al., 2011), retaining 13,708 SNPs found in 90% of all individuals with a minimum quality value of 30, and with 20–200× read coverage. We separated the VCF into mitochondrial SNPs (mtDNA) and nuclear SNPs (nDNA) by aligning sequences to the *H. castro* mitochondrial genome (Antaky et al., 2019) using bwa-mem (Li, 2013), samtools (Li et al., 2009), and vcftools (Danecek et al., 2011). We calculated fixation indices (*F*_st_) for both nDNA and mtDNA by using the Weir & Cockerham (1984) unbiased calculation in vcftools (Danecek et al., 2011). We analyzed the nDNA and mtDNA separately in R (R Core Team, 2013) using the package ‘PCAdapt’ to run a principal components analysis. We transformed the trimmed alignments in PGDSpider (Lischer & Excoffier, 2012) and ran them in STRUCTURE (Pritchard, Stephens & Donnelly, 2000) to identify the likely number of populations from which the samples came and infer proportion of ancestry for each individual. We ran STRUCTURE for one, two, three, and four populations (*K*), with 10 iterations for each K at a burn-in period of 10,000 steps and 10,000 steps after burn-in and 10 iterations at a burn-in period of 100,000 steps and 100,000 steps after burn-in. We performed multiple runs to obtain better estimates of the posterior probability of each *K* value. We input results from both STRUCTURE runs into the program STRUCTURE HARVESTER (Earl & VonHoldt, 2012), to calculate the ad-hoc statistic (Δ*K*) suggested by Evanno, Regnaut & Goudet (2005) that takes into account the change in the log probability of the data between increasing numbers of clusters. We used TASSEL (Bradbury et al., 2007) to determine the nucleotide diversity (π), *Watterson estimator* of diversity (θ), and Tajima’s *D* (*D*_T_). For population statistics, individuals were grouped by island from which they were sourced from.

We constructed a Bayesian Skyline Plot, a coalescent-based graphical method, in BEAST v.2.5.0 (Bouckaert et al., 2014) and TRACER v.1.6 (Rambaut et al., 2016) using mtDNA from all individuals to infer potential historical fluctuations in effective population size (*N*_e_). The Bayesian Skyline Plot framework takes into account genealogy, demographic history, and substitution-model parameters in a single analysis (Ho & Shapiro, 2011). We ran the Bayesian Skyline Plot analysis using strict clock models as it is considered a good approximation for intra-population level analyses and implemented in other studies on seabird evolution (Younger et al., 2015; Iglesias-Vasquez et al., 2017). We used the molecular clock rate of 1.94 × 10^−8^ substitutions/site/year which was calibrated from Hydrobatidae (Storm Petrel) mtDNA using only *Hydrobates* spp. (Weir & Schluter, 2008).

## Results

From our 24 individuals (nine samples from Kaua‘i, two from O‘ahu, two from Maui, and 11 from the Big Island) we obtained a total of 650,048,040 sequences. We calculated genetic diversity statistics using 13,708 genomic SNPs shared among all individuals. The average nucleotide diversity (π) was similar across individuals found on Kaua‘i (0.159), O‘ahu (0.197), Maui (0.172), and Big Island (0.176). The Watterson estimator of expected genetic diversity at equilibrium (θ) was higher for Maui (0.376) and O‘ahu (0.333) with the caveat of low sample size, but lower for Kaua‘i (0.193) and the Big Island (0.225) where the sample size was higher. Tajima’s *D* (*D*_T_) was negative for all island colonies (Table 2). The inbreeding coefficient (*F*_IS_) ranged from −0.354 to 0.097 across islands.

**Table 2:** Summary statistics by island for *Hydrobates castro* genetic variation across the Main Hawaiian Islands based on 13,708 gDNA Single Nucleotide Polymorphisms (SNPs).

Island	*n*	θ	*D*_T_	*F*_IS_	π
Kaua‘i	9	0.193	−0.937	−0.057	0.159
O‘ahu	2	0.376	−5.807	−0.341	0.197
Maui	2	0.333	−5.829	−0.354	0.172
Big Island	11	0.225	−1.128	0.097	0.176

**Notes:**

Due to small sample size, results should be interpreted with caution.

*n*, sample size; θ, Watterson estimator; *D*_T_, Tajima’s D; *F*_IS_, Inbreeding coefficient; π, nucleotide diversity.

We calculated pairwise *F*_st_ values between island-scale groupings of individuals using both nDNA and mtDNA. We identified 13,641 nuclear and 67 mitochondrial shared SNPs among all individuals. Based on nDNA among islands, the highest differentiation was found between two groupings of islands (Supplemental Material S1): individuals on Maui differed from those on Kaua‘i and Big Island, and individuals on O‘ahu differed from those on Kaua‘i and Big Island. The lowest *F*_st_ value was between the Maui and O‘ahu individuals, although these locations were only represented by two individuals and must be interpreted cautiously. *F*_st_ values based on mtDNA among islands followed a similar pattern, showing differentiation between the same two groupings of islands (Supplemental Material S1). The Maui and O‘ahu island samples showed no differentiation within nDNA (*F*_st_ = 0), and therefore we performed the same analyses combining the Maui and O‘ahu individuals into a single population, which did not produce qualitatively different results or alter conclusions (Supplemental Material S2).

**Figure 2:**
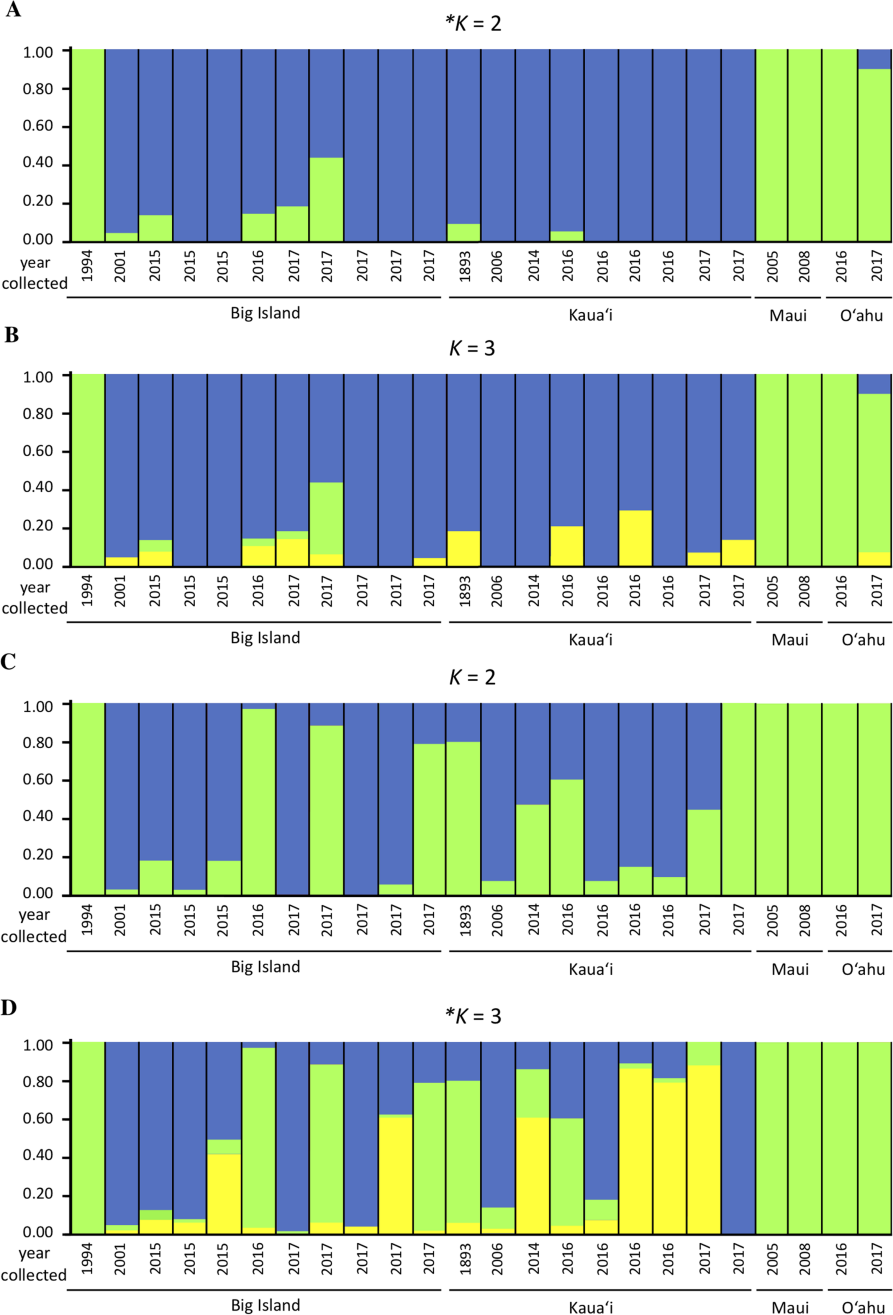
Genetic structure plots indicating inferred proportion of ancestry of *Hydrobates castro*. Plots are based on analysis of 13,641 nuclear SNPs for *K* = 2 (A) and *K* = 3 (B), and 67 mitochondrial SNPs for *K* = 2 (C) and *K* = 3 (D) using STRUCTURE. Each bar represents an individual bird and the color represents the assignment probability to a particular genetic group. The most likely clustering for *K*, denoted with an asterisk, was determined by STRUCTURE HARVESTER.

**Figure 3:**
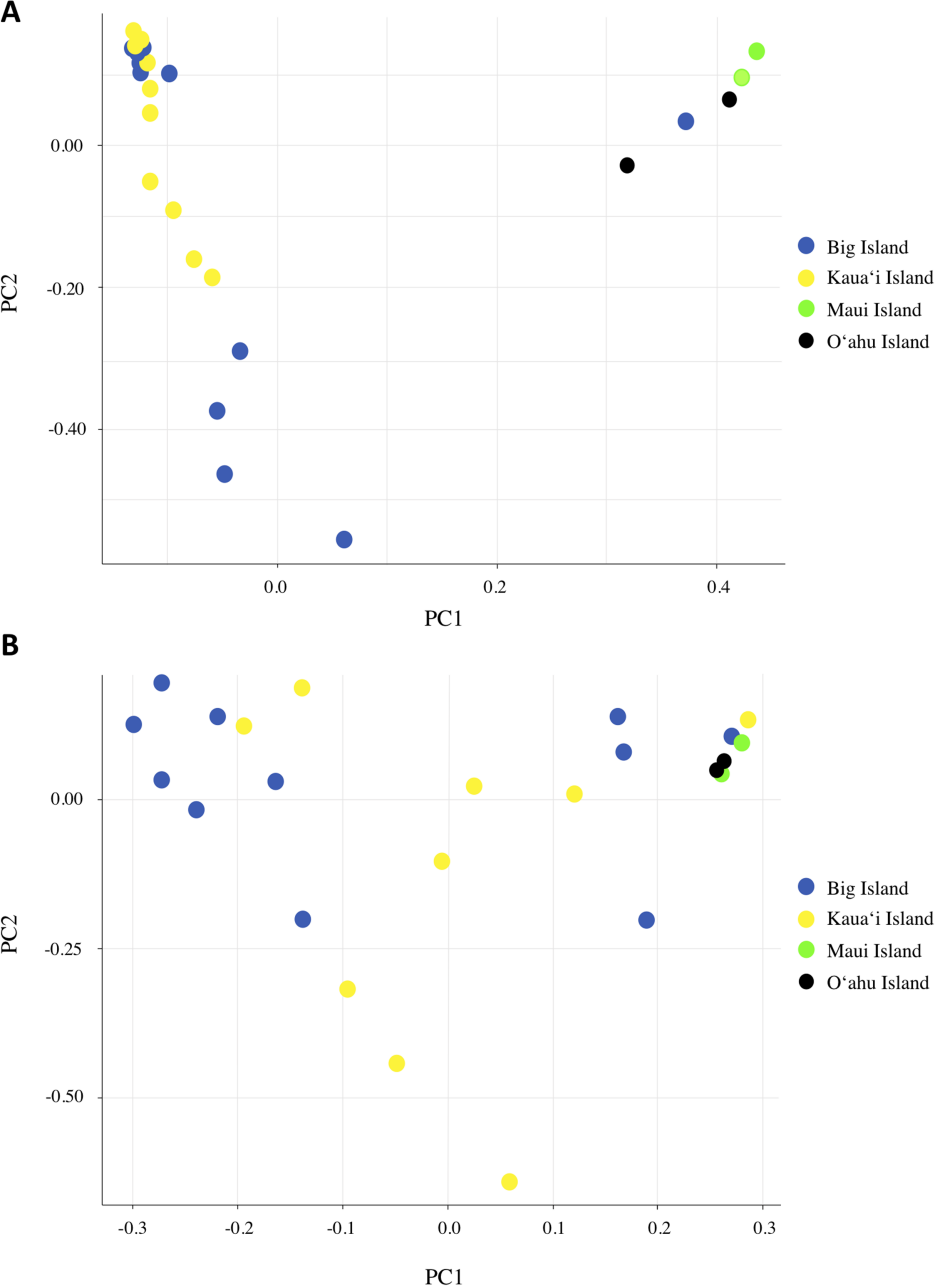
Principal component analysis based on (A) nDNA variation and (B) mtDNA variation for *Hydrobates castro*. Each dot represents a single bird with colors indicating where the bird was found across the Main Hawaiian Islands.

We investigated population structure using both nDNA and mtDNA with a Bayesian clustering approach. Using the criteria of Evanno, Regnaut & Goudet (2005), the grouping *K* = 2 received the highest support for nDNA and the grouping *K* = 3 received the highest support for mtDNA (Supplemental Material S3). Although the Evanno criteria cannot calculate the likelihood for *K* = 1, due to the separation of the Maui/O‘ahu group and Big Island/Kaua‘i in the *K* = 2 structure plot and PCA plot, it is unlikely that all individuals tested fall into a single genetic population. Furthermore, the Maui/O’ahu group had a 0.977 average probability of assigning together while the Big Island/Kaua’i group showed a 0.904 average probability of assigning to the same cluster. The structure analysis from the nDNA shows some support for separation of populations by island (Fig. 2A), which is consistent with philopatry of breeding individuals. We evaluated relationships among individuals using a principal component analysis (PCA). The PCA based on nDNA resulted in at least two groupings, with some indication of separation by island, the same pattern as the structure analysis (Fig. 3A). The PCA and structure analysis based on mtDNA did not show the same separation of population by island as seen in the nDNA (Figs. 2D and 3B).

Reconstruction of the population size history by means of the coalescent Bayesian skyline plot using mtDNA data suggests a likely continuous population decline over the last 500 years for *H. castro* with indication of a small expansion in the last 25 years (Supplemental Material S4). Although the general trend in the skyline plot shows a decline, the median effective population number stays relatively stable with broad confidence intervals indicating that a constant population size over the whole time period is also possible. The current effective population size (*N*_e_) ranged from 166 to 2327 individuals with a mean of 414. As the Bayesian skyline plot assumes that there is panmixia within the population tested, the four individuals found on Maui and O‘ahu were removed from this analysis as they may belong to a different genetic group. Furthermore, results from this analysis should be interpreted with caution as the SNP data may differ from the overall mutation rate for mtDNA, as the more slowly evolving sites within mtDNA are likely not fully represented.

## Discussion

In this study, we found that *H. castro* in the Hawaiian Islands had relatively low inbreeding estimates and high genetic diversity, despite a relatively small population size and an assumed high degree of philopatry based on taxonomic order (Antaky et al., in press). Individuals from the presumed breeding colonies on the Big Island and Kaua‘i show little differentiation, but individuals recovered from Maui and O‘ahu do not assign to breeding colonies on either the Big Island or Kaua‘i island, suggesting the presence of another distinct population in the region.

Analysis of Hawaiian populations of *H. castro* using SNP data indicates an excess of rare alleles (mean *D*_T_ = −3.425 ± 2.76), low rates of inbreeding (mean *F*_IS_ = −0.164 ± 0.221), and high nucleotide diversity (mean π = 0.176 ± 0.016). Although the species has undergone a decline in population size, there is no evidence of inbreeding, potentially due to promiscuous mating behavior or sex-biased dispersal (Greenwood, 1980; Amos et al., 2001; Milot et al., 2007). Nucleotide diversities of *H. castro* in the Hawaiian Islands were higher than those found in studies of some non-endangered seabird species using RADseq methods (Dierickx et al., 2015; Tigano et al., 2017) but not all (Clark, 2018). The Bayesian skyline plot suggests a steady population decline after the introduction of mammalian predators to the Hawaiian Islands, with a small expansion in the last 25 years, but due to small sample size and assumptions of the Bayesian skyline analysis, evidence of recent population size change is relatively weak (Supplemental Material S4). Past studies estimated the breeding population of *H. castro* to be 660 individuals based on radar surveys, observational records, and song meter data (Pyle & Pyle, 2017), which falls within the 95% confidence intervals for effective population size found in this study (127–1,559). While these results are consistent, comparisons of census and effective population size are notoriously difficult (Frankham, 1995; Turner, Wares & Gold, 2002), and further studies investigating population size are necessary as skyline plots are not appropriate for the inference of complete historical demography (Grant, 2015).

Due to the nature of cryptic Endangered species with low population numbers, there are potential introduced biases from small sample size and opportunistic sampling. We acknowledge that with reduced sampling, we risk not capturing the complete genetic diversity in the populations sampled. Thus, additional studies will be useful to validate the results suggested from this study. Whole genome amplification was used to increase DNA yield in samples. Although whole genome amplified DNA for next-generation sequencing and array applications have been debated, studies using Qiagen’s REPLI-g kits to amplify DNA yield have proven to be effective in limiting amplification bias across alleles and producing comparable results to the non-amplified sequence (Cichon et al., 2008; Ahsanuddin et al., 2017). Due to inaccessibility and lack of knowledge of nest site locations, sampling cannot be performed on confirmed breeding adults in the field. It is possible that carcasses found at or near colonies may be juveniles or adults visiting from other islands or populations. To limit this bias, we sequenced each individual and performed analyses that do not assume population assignment (PCA, STRUCTURE). However, for population diversity statistics and *F*_st_ analysis (Table 2; Supplemental Material S1), which rely on population allele frequencies, we note that results should be interpreted with caution due to small sample sizes.

Based on the structure analysis and PCA, we found evidence for at least two distinct groups, with individuals from Kaua‘i and the Big Island grouping together and individuals from Maui and O‘ahu islands not assigning to that same population (Figs. 2A and 3A). These genetic patterns do not match the island chain’s geography, and instead may be due to genetic drift in small fragmented colonies, ocean regime around the islands (Friesen, 2015), or that the individuals collected on Maui and O‘ahu were visiting birds that may belong to another distinct breeding population outside Hawai‘i. In sum, the Maui and O‘ahu island individuals differ from the individuals breeding on Kaua‘i and the Big Island. This is further supported by the same analyses performed with Maui and O‘ahu sourced individuals combined together (Supplemental Material S2). Due to small sample size, however, our results should be interpreted with caution. The PCA and structure analysis based on mtDNA did not show the same pattern of separation as nDNA, possibly because mtDNA does not account for male-mediated dispersal, or because the mtDNA dataset had fewer SNPs included in the analysis.

With some indication of differences among islands in the structure analysis and PCA plots, genetic data are consistent with the expectation that *H. castro* is a highly philopatric species. While Procellariiformes are more likely to return to their natal colony than disperse, most species are not completely philopatric, and a small percentage of individuals are likely to disperse to new colonies (Antaky et al., in press). It only takes a small amount of dispersal, that is, less than ten migrants per generation, to homogenize genetic structure (Mills & Allendorf, 1996). Seabirds also mate while on visiting forays to neighboring colonies, increasing gene flow beyond that expected based on dispersal from the natal colony (Young, 2010). The individual sampled in 1994 on the Big Island (Table 1), which clustered with the Maui/O‘ahu group in both the PCA and structure analysis, is most likely a migrant from the Maui/O‘ahu group that was visiting or migrated to breed on the Big Island. Thus, complex population structure must be taken into account when interpreting population genetics in highly mobile species (Bowen et al., 2005).

Relatively high genetic diversity despite population declines has been observed in other long-lived endangered seabird species (e.g., the Hawaiian Petrel *Pterodroma sandwichensis*, Welch et al., 2012; the Balearic Shearwater *Puffinus mauretanicus*, Genovart et al., 2007; the Magenta Petrel *Pterodroma magenta*, Lawrence et al., 2008), and may be explained by evolutionary history. An ancient large population of *H. castro* may lead to retained genetic diversity (Goossens et al., 2005). Despite a likely population decline since the introduction of nonnative mammalian predators to the Hawaiian Islands within the last 1,100 years (Pyle & Pyle, 2017; this manuscript), relatively high genetic diversity in *H. castro* is not completely unexpected as only a few hundred individuals may be needed to maintain a majority of genetic diversity (Gaither et al., 2010; Tison, 2014).

## Conclusions

This study found little population structure between Kaua‘i and the Big Island, and no inbreeding within the Hawaiian populations of *H. castro*, indicating that at least some individuals are dispersing among the breeding colonies on these islands to maintain gene flow. However, the fact that bycatch birds from Maui and O‘ahu do not assign to the same breeding colony as Kaua‘i and the Big Island also supports the existence of a second discrete population in the Hawaiian Islands or possibly outside of Hawai‘i (e.g., Japan). Although a lack of detection at the suspected Maui Nui breeding colony precludes direct testing, this island may host a breeding colony distinct from the others (United States Fish and Wildlife Service, 2016), or there may be unknown temporal separation of nesting populations in the Hawaiian Islands that have yet been tested (Raine et al., 2017) similar to that reported for *H. castro* in Cape Verde (Monteiro & Furness, 1998; Deane, 2013). Continued efforts to find active colonies in the Hawaiian Islands are essential to assess population connectivity and for species recovery (Young et al., 2019).

Populations of *H. castro* currently do not appear to be in any danger of a genetically induced extinction vortex (Gilpin & Soulé, 1986). However, they remain vulnerable to other threats (Jones et al., 2008; Croxall et al., 2012; Spatz et al., 2014). Reduced fledging success and adult mortality due to invasive predators continue to impact population growth (Galase, 2019). Predator control, translocation, and related management efforts to increase chick survival, attract conspecifics, help expand colony range, minimize adult mortality, and increase nesting success will be crucial in achieving recovery in this species (Raine et al., 2017; Antaky, Galase & Price, 2019).

In summary, despite a historical population decline, continued small population size, and separation of hundreds of miles among islands, this study finds no evidence that populations of *H. castro* in the Hawaiian Islands are inbred. *H. castro* colonies in Hawai‘i appear to have escaped any severe genetic bottleneck, and the populations do not seem at risk for an extinction vortex associated with loss of genetic diversity (Gilpin & Soulé, 1986). Nevertheless, the small population size of *H. castro* warrants continued conservation programs to achieve recovery, as seabirds play an important role in food webs in both marine and terrestrial ecosystems in the Pacific (Hobson, Piatt & Pitocchelli, 1994; Fukami et al., 2006) and hold cultural significance to Hawaiian communities (Kamakau, 1987; Rose, Conant & Kjellgren, 1993; United States Fish and Wildlife Service, 2005; National Park Service, 2006).

## Supplemental Information

10.7717/peerj.8463/supp-1 Supplemental Information 1 Supplementary Material S1–S4. Supplementary Material S1: Estimates of FST from mtDNA data (light gray) and FST from nDNA (dark gray) for pairwise comparisons between *Hydrobates castro* grouped by sourced island across the Main Hawaiian Islands, where *n* = sample size.
Supplementary Material S2: (A) Fst estimates (B) structure plot, and (C) PCA plot, using nDNA and assigning the Maui and O‘ahu sourced individuals together as all belonging to the Maui island population.
Supplementary Material S3: K statistics (Evanno, Regnaut & Goudet, 2005) using (A) nDNA and (B) mtDNA of *Hydrobates castro* for STRUCTURE runs with the number of clusters (K) set between 1 and 4. Delta K statistic takes into account the change in the log probability of the data as K increases, but it cannot be calculated for *K* = 1 which is also a potential answer. The highlighted row is the chosen K with the highest support from STRUCTURE HARVESTER.
Supplementary Material S4: Bayesian Skyline Plot of *Hydrobates castro* based on the 67 mitochondrial SNPs from 20 samples collected in Kaua‘i and Big Island. The black line represents the median of the parameter Ne, proportional to the effective population size, while the purple shading is the 95% CI.

10.7717/peerj.8463/supp-2 Supplemental Information 2 SNP dataset. The SNP dataset includes VCF files of the filtered nuclear and mitochondrial SNPs shared between *Oceanodroma castro* in the Hawaiian Islands.

## Additional Information and Declarations

### Field Study Permissions

The following information was supplied relating to field study approvals (i.e., approving body and any reference numbers): We collected samples under US Fish & Wildlife Permit Number TE25955C-0 and State of Hawaii Department of Land & Natural Resources Protected Wildlife Permit Number WL19-01.

### Data Availability

The following information was supplied regarding data availability: The SNP dataset and contigs used for analysis are available in the Supplemental Files. Raw sequence data is available at NCBI SRA: PRJNA559669. The Band-rumped Storm Petrel samples (tissue/feather/blood) are stored in the Hawaiʻi Wildlife Ecology Lab at the University of Hawaiʻi at Mānoa. The sampled museum specimens used in this study are stored at the Bernice Pauahi Bishop Museum: 156975, 185162, 185001, 185313, 183608, 184416.
