# Supplementary material for: Unexpectedly high genetic diversity in a rare and endangered seabird in the Hawaiian Archipelago
Source: PeerJ. 2020 Feb 6;8:e8463. doi: 10.7717/peerj.8463 (PMC7007978; doi:10.7717/peerj.8463)
Supplement: Supplemental Information 1 — Supplementary Material S1: Estimates of FST from mtDNA data (light gray) and FST from nDNA (dark gray) for pairwise comparisons between Hydrobates castro grouped by sourced island across the Main Hawaiian Islands, where n = sample size. Supplementary Material S2: (A) Fst estimates (B) structure plot, and (C) PCA plot, using nDNA and assigning the Maui and O‘ahu sourced individuals together as all belonging to the Maui island population. Supplementary Material S3: K statistics (Evanno, Regnaut & Goudet, 2005) using (A) nDNA and (B) mtDNA of Hydrobates castro for STRUCTURE runs with the number of clusters (K) set between 1 and 4. Delta K statistic takes into account the change in the log probability of the data as K increases, but it cannot be calculated for K = 1 which is also a potential answer. The highlighted row is the chosen K with the highest support from STRUCTURE HARVESTER. Supplementary Material S4: Bayesian Skyline Plot of Hydrobates castro based on the 67 mitochondrial SNPs from 20 samples collected in Kaua‘i and Big Island. The black line represents the median of the parameter Ne, proportional to the effective population size, while the purple shading is the 95% CI. [file peerj-08-8463-s001.docx]

SUPPLEMENTARY MATERIAL

**Unexpectedly high genetic diversity in a rare and Endangered seabird in the Hawaiian Archipelago**

Carmen C. Antaky*, Emily E. Conklin, Robert J. Toonen, Ingrid S. Knapp, & Melissa R. Price

*Corresponding author: antaky@hawaii.edu

**Supplementary Material S1:** Estimates of *F_ST_* from mtDNA data (light gray) and *F_ST_* from nDNA (dark gray) for pairwise comparisons between *Hydrobates castro* grouped by sourced island across the Main Hawaiian Islands, where n = sample size.

| **Island** | n | Kauaʻi | O‘ahu | Maui | Big Island |
| --- | --- | --- | --- | --- | --- |
| Kauaʻi | 9 | - | 0.1647 | 0.1654 | 0.0073 |
| O‘ahu | 2 | 0.2068 | - | 0.3000 | 0.2720 |
| Maui | 2 | 0.2244 | 0.0000 | - | 0.2625 |
| Big Island | 11 | 0.0015 | 0.1295 | 0.1498 | - |

*Due to small sample size, results should be interpreted with caution.

**Supplementary Material S2:** **(A)** *F_st_* estimates, **(B)** structure plot, and **(C)** PCA plot, using nDNA and assigning the Maui and O‘ahu sourced individuals together as all belonging to the Maui island population.

**A**

| **Island** | n | Kauaʻi | Maui/O‘ahu | Big Island |
| --- | --- | --- | --- | --- |
| Kauaʻi | 9 | - |  |  |
| Maui | 4 | 0.3166 | - |  |
| Big Island | 11 | 0.0015 | 0.1588 | - |

**B
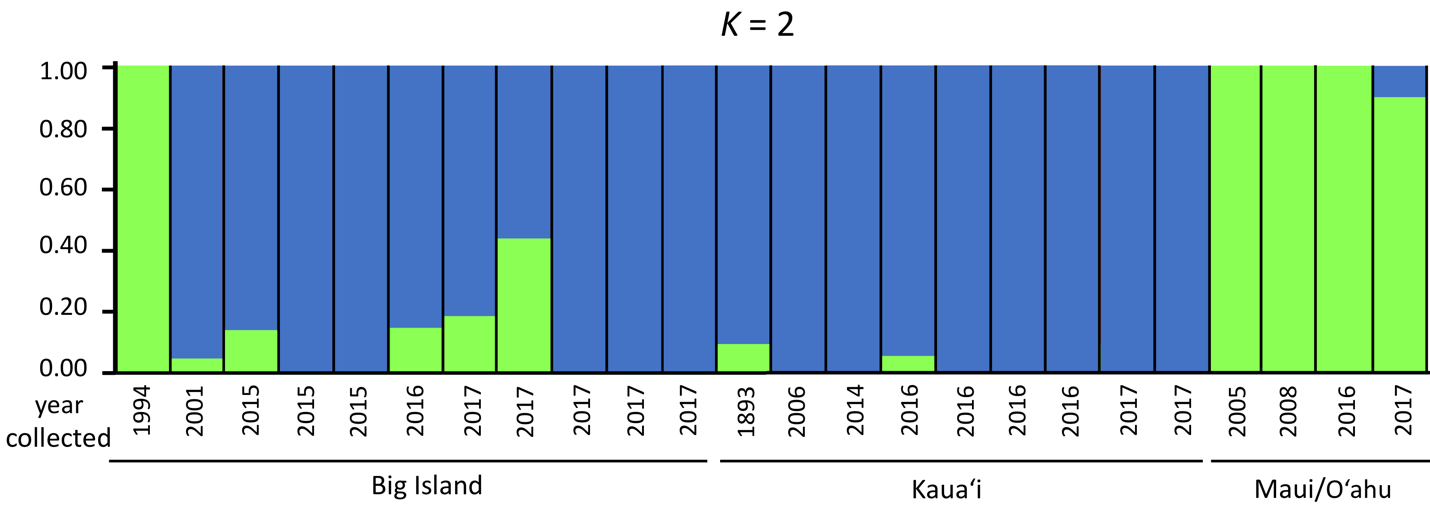
**

**C**

**
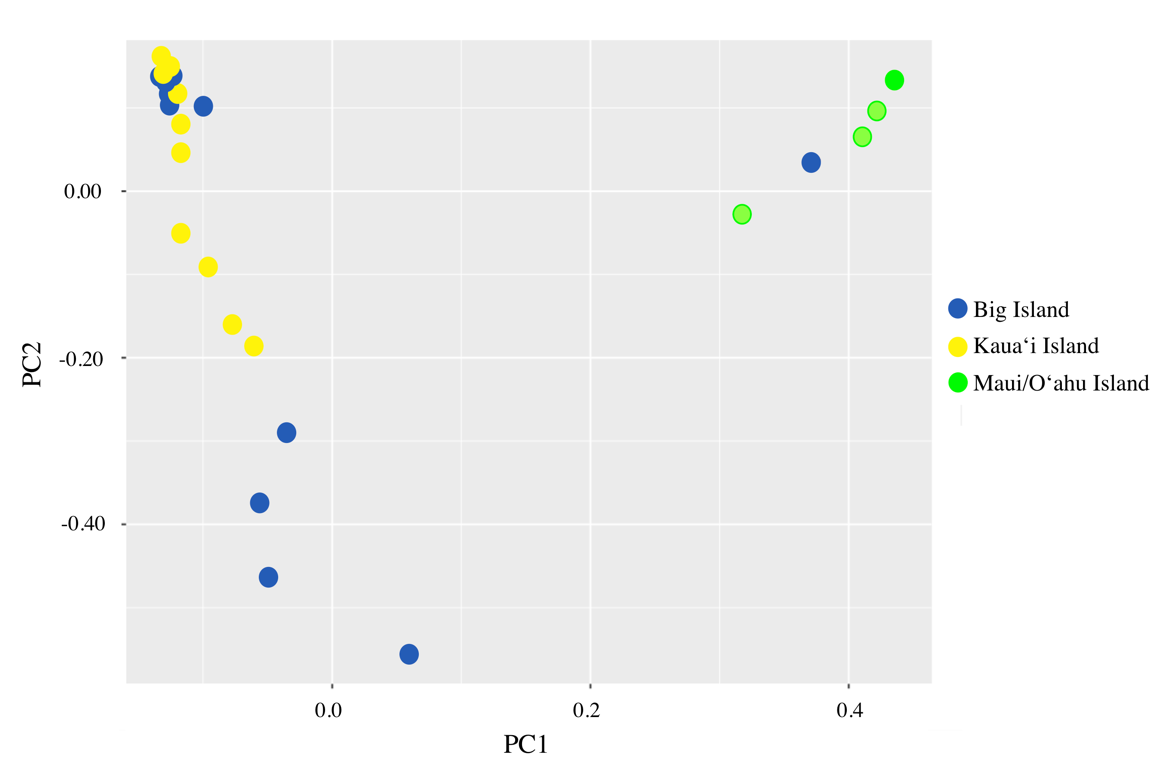
**

**Supplementary Material S3:** *K* statistics (Evanno et al. 2005) using (**A)** nDNA and **(B)** mtDNA of *Hydrobates castro* for STRUCTURE runs with the number of clusters (*K*) set between 1 and 4. Delta *K* statistic takes into account the change in the log probability of the data as *K* increases, but it cannot be calculated for K=1 which is also a potential answer. The highlighted row is the chosen *K* with the highest support from STRUCTURE HARVESTER.

| **K** | **Mean LnP(K)** | **StDevP(K)** | **Ln’** | **\|Ln”(K)\|** | **Delta K** |
| --- | --- | --- | --- | --- | --- |
| 1 | -162182.19 | 56.51 | – | – | – |
| 2 | -138540.84 | 147.71 | 23641.35 | 23119.61 | 156.52 |
| 3 | -138019.10 | 198.74 | 521.74 | 616.59 | 3.102 |
| 4 | -138113.95 | 189.11 | -94.85 | – | – |

**A**

**
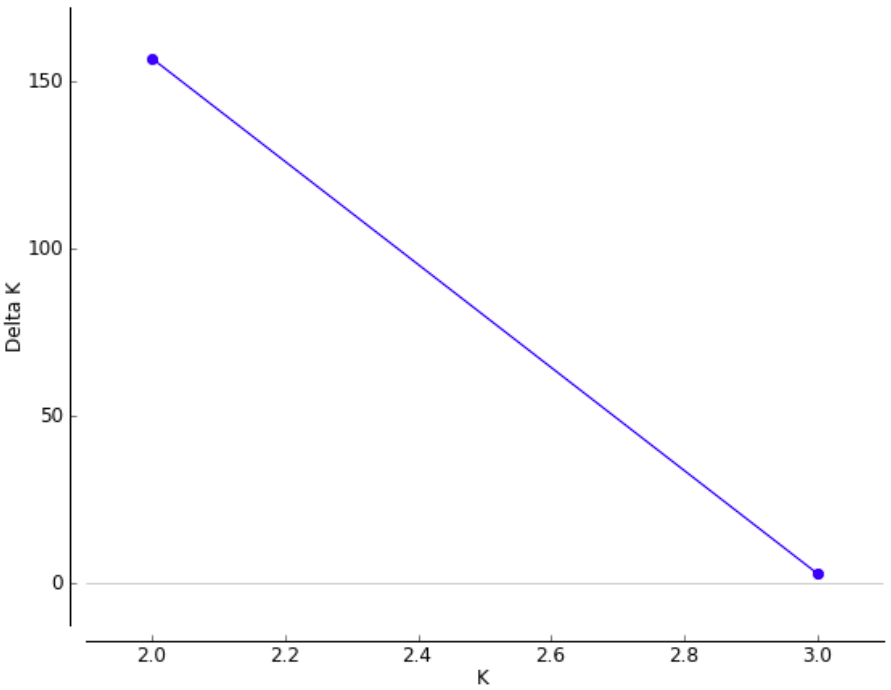
**

| **K** | **Mean LnP(K)** | **StDevP(K)** | **Ln’** | **\|Ln”(K)\|** | **Delta K** |
| --- | --- | --- | --- | --- | --- |
| 1 | -923.03 | 0.58 | – | – | – |
| 2 | -610.40 | 31.97 | 312.63 | 260.14 | 8.14 |
| 3 | -557.92 | 1.31 | 52.49 | 84.89 | 64.98 |
| 4 | -590.32 | 27.69 | -32.41 | – | – |

**B**

**
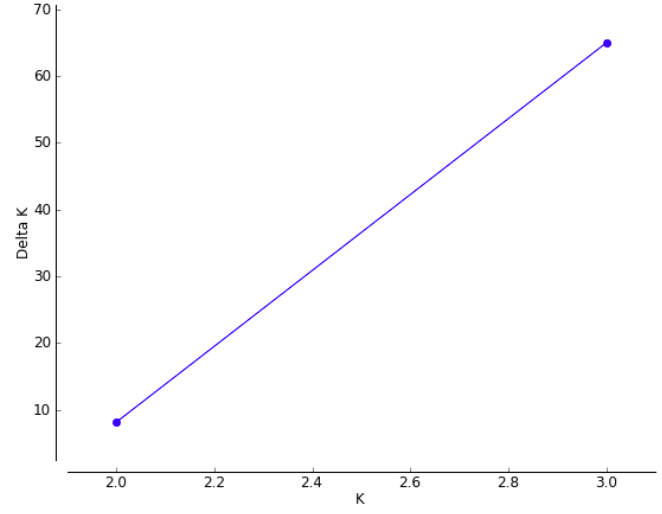
**

**Supplementary Material S4:** Bayesian Skyline Plot of *Hydrobates castro* based on the 67 mitochondrial SNPs from 20 samples collected in Kaua‘i and Big Island. The *black line* represents the median of the parameter N_e_, proportional to the effective population size, while the *purple shading* is the 95 % CI.

**
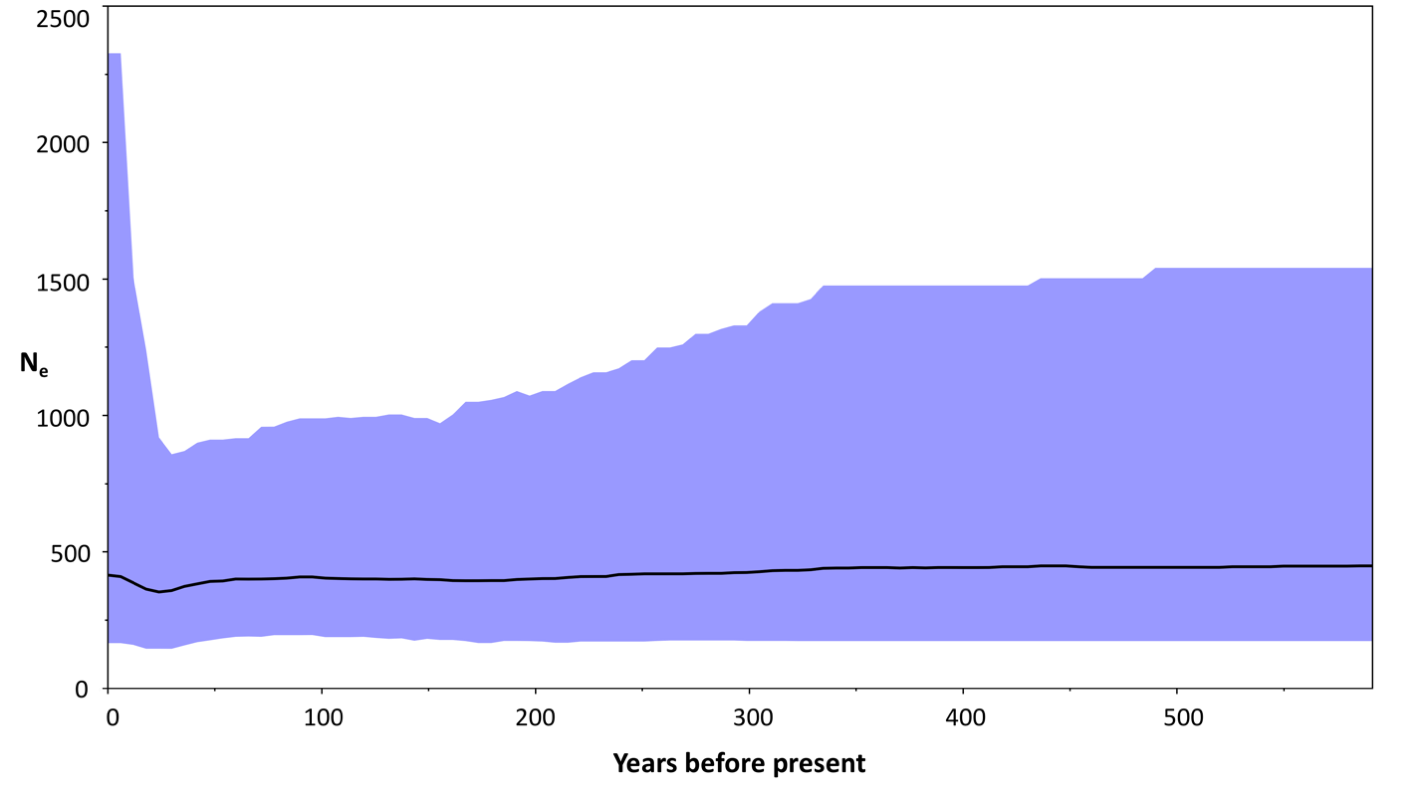
**

*Due to small sample size, results should be interpreted with caution.
